# Radiotherapy plus nimotuzumab or placebo in the treatment of high grade glioma patients: results from a randomized, double blind trial

**DOI:** 10.1186/1471-2407-13-299

**Published:** 2013-06-19

**Authors:** Maria Teresa Solomón, Julio César Selva, Javier Figueredo, José Vaquer, Carolina Toledo, Nelson Quintanal, Silvia Salva, Rafael Domíngez, José Alert, Jorge Juan Marinello, Mauricio Catalá, Martha González Griego, Juan Antonio Martell, Patricia Lorenzo Luaces, Javier Ballesteros, Niurys de-Castro, Ferdinand Bach, Tania Crombet

**Affiliations:** 1 Calixto García Hospital, Havana, Cuba; 2 Lucía Iñiguez Hospital, Holguin, Cuba; 3 Center for Medical and Surgical Research, Havana, Cuba; 4 Arnaldo Milián Hospital, Santa Clara, Cuba; 5 Maria Curie Hospital, Havana, Cuba; 6 Luis Díaz Soto Hospital, Havana, Cuba; 7 Hermanos Ameijeiras Hospital, Havana, Cuba; 8 Saturnino Lora Hospital, Santiago de Cuba, Cuba; 9 National Institute of Oncology and Radiobiology, Havana, Cuba; 10 National Center for Clinical Trials, Havana, Cuba; 11 Center of Molecular Immunology, PO BOX 16040, Havana 11600, Cuba; 12 University of the Basque Country, Havana, Spain; 13 Institute of Pharmacy and Food, Havana, Cuba; 14 Oncoscience AG, Wedel, Germany

**Keywords:** High grade glioma (HGG), Nimotuzumab, EGFR, Monoclonal antibody, Adult glioma, Anaplastic astrocytoma, Glioblastoma multiforme

## Abstract

**Background:**

The prognosis of patients bearing high grade glioma remains dismal. Epidermal Growth Factor Receptor (EGFR) is well validated as a primary contributor of glioma initiation and progression. Nimotuzumab is a humanized monoclonal antibody that recognizes the EGFR extracellular domain and reaches Central Nervous System tumors, in nonclinical and clinical setting. While it has similar activity when compared to other anti-EGFR antibodies, it does not induce skin toxicity or hypomagnesemia.

**Methods:**

A randomized, double blind, multicentric clinical trial was conducted in high grade glioma patients (41 anaplastic astrocytoma and 29 glioblastoma multiforme) that received radiotherapy plus nimotuzumab or placebo. Treatment and placebo groups were well-balanced for the most important prognostic variables. Patients received 6 weekly doses of 200 mg nimotuzumab or placebo together with irradiation as induction therapy. Maintenance treatment was given for 1 year with subsequent doses administered every 3 weeks. The objectives of this study were to assess the comparative overall survival, progression free survival, response rate, immunogenicity and safety.

**Results:**

The median cumulative dose was 3200 mg of nimotuzumab given over a median number of 16 doses. The combination of nimotuzumab and RT was well-tolerated. The most prevalent related adverse reactions included nausea, fever, tremors, anorexia and hepatic test alteration. No anti-idiotypic response was detected, confirming the antibody low immunogenicity. The mean and median survival time for subjects treated with nimotuzumab was 31.06 and 17.76 vs. 21.07 and 12.63 months for the control group.

**Conclusions:**

In this randomized trial, nimotuzumab showed an excellent safety profile and significant survival benefit in combination with irradiation.

**Trial registration:**

Cuban National Register for clinical trials (No. 1745) (http://registroclinico.sld.cu/ensayos).

## Background

High-grade gliomas (HGG) are the most common primary tumors in the central nervous system (CNS) in adults [1]. Despite remarkable advances in cancer research and in neurosurgery, radiotherapy and chemotherapy, these patients still face a poor prognosis, pointing towards an urgent need for new therapeutic approaches [2]. Standard treatment for HGG usually entails surgery followed by radiotherapy plus chemotherapy. Temozolomide is the drug of choice since 2005 for glioblastoma multiforme (GBM) patients [3], but unfortunately, it is not available in Cuba, due to the commercial restrictions imposed by the US embargo. However, since the survival benefit of radio-chemotherapy is so limited [4], patients with brain tumors are considered candidates for clinical trials that evaluate new drugs, radiosensitizers or new accelerated/hyperfractionated radiation schemes. Therefore, we decided to evaluate the efficacy of radiation plus an anti-EGFR antibody vs. radiation plus placebo in a controlled double blind trial, in newly diagnosed patients with grade III/IV astrocytomas.

The Epidermal Growth Factor Receptor (EGFR) is a membrane-bound receptor that has been shown to have a major role in the pathogenesis and progression of different cancers [5]. EGFR is greatly expressed in HGG patients and gene amplification represents one of the most frequent alterations in this tumor type [6]. Moreover, EGFR plays a fundamental role in gliomagenesis. According Mazzoleni and co-workers, cancer stem cells (CSC) isolated from glioma patients, need to express EGFR to promote experimental tumorigenesis and EGFR-expressing initiating cells display the most malignant phenotype [7]. In summary, EGFR is well validated as a primary contributor of HGG initiation and progression [8].

Nimotuzumab is a humanized monoclonal antibody that recognizes the EGFR extracellular domain. The antibody was obtained by humanization of the murine antibody ior egf/r3 [9]. Because nimotuzumab has a 10 fold lower affinity to the EGFR, as compared to cetuximab, its capacity to bind EGFR is heavily dictated by cell receptor density [10]. Nimotuzumab preclinical and clinical characterizations have been summarized before [11-13].

A distinguishing feature of nimotuzumab compared to other mAbs of the EGFR class, is the lack of severe skin toxicity as well as severe hypomagnesemia [14]. Two hypotheses have been posed to explain this lack of skin toxicity of nimotuzumab: according Garrido [10], nimotuzumab requires bivalent binding for stable attachment to the cellular surface, leading to selectively binding to cells that express moderate to high EGFR levels. Accordingly, nimotuzumab will selectively target tumors, and not normal tissues. Instead, Talavera built a computer model of the nimotuzumab-EGFR complex [15], where nimotuzumab blocks ligand binding, but allows the receptor to adopt its active conformation, warranting the basal level of signaling needed for the survival of non-tumor cells [15].

This type of binding is analogous to the binding of trastuzumab to the HER-2 receptor [16]. Nimotuzumab safety profile permits up to 800 mg doses in adults [17] or 150–250 mg/m2 in children, without safety concerns [13].

In the nonclinical setting, nimotuzumab has been evaluated in the glioma cell line U87MG. Co-administration of the antibody with radiation increased the radiosensitivity, resulting in a delay of tumor growth. The antibody reduced angiogenesis and the total number of radioresistant cancer stem cells [18].

In a separate study, nude mice that had an intra-cerebral implant of the U87MG cell line were treated with nimotuzumab labelled with ^111^Indium. Radioactivity was measured after organ explantation. Results showed a clear time-dependant increase in ^111^indium nimotuzumab uptake in the tumour in contrast to all other organs [19].

The capacity of the antibody of crossing the blood–brain barrier (BBB) was studied also by radioimmunscitigraphy using nimotuzumab labelled with Technetium 99 (Tc99). In a phase I/II trial, immunscitigraphy done after radiation plus nimotuzumab, showed a positive MAb uptake by patients with tumors, while subjects with complete responses showed no antibody accumulation at the known site of tumors [20]. In addition, it has been proposed that in the fast-growing gliomas, the newly formed blood vessels lack BBB function. As a consequence, MAbs such as nimotuzumab may primarily enter a brain tumor through tumor vessels that lack BBB [21,22].

To conclude, the nonclinical and clinical radiolabelled study does support penetration of the brain. MRI scan results in children with refractory brain tumors treated with nimotuzumab alone also provided evidence of nimotuzumab activity at the tumor site [13].

An open labeled study of the combination nimotuzumab plus radiotherapy (RT)/temozolomide (TMZ) study was conducted in Germany in 149 adult patients with newly diagnosed GBM [23]. In this paper, we report the results of a randomized, double blind clinical trial where 70 HGG patients were treated with irradiation plus nimotuzumab or placebo.

## Methods

A randomized, double-blind, multicentric, Phase II clinical trial was conducted in 70 HGG patients that received irradiation plus either nimotuzumab or a placebo. Sample size was calculated by anticipating a 6-month improvement in median survival time with respect to the baseline survival in the control group. The sample size was calculated using a “sample size calculation” software, Version 1.1, option 4: comparison of two means, from the Institute of Medical Research in Barcelona.

Patients were recruited at 8 clinical sites. They were subjected to maximal excision (total, partial resection or biopsy) at least 4 weeks before the inclusion and were candidates of radiotherapy. Other important inclusion criteria included: age older than 18, Karnofsky performance status (KPS) ≥ 60 and adequate bone marrow, liver and renal function; subjects in fertile age were required to possess a negative pregnancy test and to use an effective contraceptive method. The primary objective of this study was to assess the overall survival (OS) of nimotuzumab in HGG patients when compared to the control group receiving irradiation and placebo. The secondary objectives were to assess progression-free survival (PFS), response rate and the safety and immunogenicity of nimotuzumab in this patient population. Eligible patients were randomized to either group in a 1:1 ratio.

Random assignment was performed centrally through a validated simple randomization system version 1.2. Patients were previously stratified by histology (GBM vs. AA) to ensure equal distribution in both groups. The study was designed to include up to 30 GBM patients while the rest were AA patients. Treatment dose was 200 mg of nimotuzumab, intravenously infused over 30 to 60 minutes. The control group received 4 vials of a placebo composed by a saline buffer. Each subject received a weekly infusion, for 6 weeks, concurrently with the radiotherapy. After finishing induction therapy, patients received, in double blind fashion, a maintenance dose of 200 mg of nimotuzumab or 4 vials of placebo every 21 days until completing 1 year of treatment.

Irradiation was delivered in doses of 180–200 cGy given once daily, 5 days per week, to a total dose of 5000 cGy to 6000 cGy. Radiotherapy planning and simulation was performed on the basis of recent CT scans. The irradiation field encompassed the initial tumor volume plus a security margin of 2 cm.

Before each dose, a physical examination of the major body systems was conducted. Vital signs were measured before and after each infusion. Hematology and biochemistry tests were carried out previous to the first dose and every 14 days for 6 weeks. Later on, sampling was carried out every 21 days, until 1 year of study.

Adverse events were assigned severity/intensity categories according the Common Terminology Criteria for Adverse Events (CTCAE) version 3. The anti-idiotypic response against nimotuzumab, was measured in 12 patients through an indirect ELISA system validated at the Center of Molecular Immunology, that has been previously described [20]. For response evaluation, nuclear magnetic resonance (MRI) or CT-scans were done before inclusion and then, every 3 months. Response was classified according to WHO modified criteria. Overall survival and progression free survival were analyzed using the Kaplan-Meier method and the parametric Weibull regression survival model [24]. The Weibull Shape Parameter (WSP) test is very powerful at detecting signals that occur shortly after starting treatment [24].

The trial was performed in compliance with the Helsinki Declaration. The protocol was approved by the Institutional Review Boards of the 8 research sites: Calixto García Hospital, Lucía Iñiguez Hospital, Center for Medical and Surgical Research, Arnaldo Milián Hospital, Maria Curie Hospital, Luis Díaz Soto Hospital, Hermanos Ameijeiras Hospital and Saturnino Lora Hospital, as well as by the National Regulatory Authority: the State Centre for Drug Quality Control. All patients signed the informed consent form. The protocol information was included on the National Register for clinical trials (No. 1745) which is a primary register approved by the World Health Organization (WHO) (http://registroclinico.sld.cu/ensayos).

## Results

A total of 73 patients were included in the study: 43 patients with Anaplastic Astrocytoma (AA) and 30 patients with Glioblastoma Multiforme (GBM). Three patients from the nimotuzumab arm (10, 54 and 61) abandoned the study from inclusion and did not receive any therapy. Information was available from 70 subjects: 41 AA and 29 GBM patients. In the AA group, 41 patients were analyzed per intention to treat: 23 received placebo and 18 received nimotuzumab. In the GBM group, 29 patients were analyzed, 15 of these received placebo and 14 received nimotuzumab.

The trial started on June 2005 and was completed on June 2010. Baseline characteristics are described in Table 1.

**Table 1:** Patient demographic and tumor characteristics

**Variables**	**Number of patients**	**Number of patients**
	**(%)**	**(%)**
	**Control group**	**Nimotuzumab patients**
Sex		
Male	19 (50%)	21 (65.6%)
Female	19 (50%)	11 (34.4%)
Race		
White	31 (81.5%)	27 (84.3%)
Black	1 (2.6%)	2 (6.3%)
Mixed	6 (15.8%)	3 (9.3%)
Age		
Mean	45.5	47.2
Median	46.5	44
Grouped age		
Younger than 50	21 (55.3%)	19 (59.4%)
Older than 50	17 (44.7%)	13 (40.6%)
Body weight		
Mean	68.1 kg	69 kg
Median	65 kg	70 kg
Histology		
AA	23 (60.5%)	18 (56.2%)
GBM	15 (39.5%)	14 (43.7%)
KPS		
100	8 (21%)	9 (28.1%)
90	9 (23.6%)	10 (31.2%)
80	6 (15.8%)	8 (25%)
70	11 (28.9%)	2 (6.2%)
60	4 (10.5%)	3 (9.3%)
Previous surgery		
Total	2 (5.4%)	5 (17.2%)
Partial	27 (72.9%)	14 (48.2%)
Biopsy	8 (21.6%)	10 (34.5%)

The groups were balanced for the most important prognostic features: histology, age, surgical intervention and KPS. In total, 32 patients received nimotuzumab and RT while 38 patients were treated with irradiation and a placebo. Nimotuzumab group received an average dose of 2631 mg, although the median cumulative dose was 3300 mg. The maximal administered dose was 3600 mg. The median number of doses was 16. Concerning radiotherapy, the mean cumulative dose was 5556 cGy.

The combination of nimotuzumab and RT was well-tolerated. More than 85% of the adverse events in either group were categorized as grade 1 or 2 (mild or moderate), according the CTCAE scale. Of these, only 15% were adverse reactions, which are, causally linked to nimotuzumab. No dose reduction was required as a consequence of an adverse event. In the placebo arm, the most frequent adverse events consisted on headache, seizures, dry radiodermitis, fever, asthenia, alopecia and alteration of the liver function tests.

In the nimotuzumab arm, the most common adverse reactions included nausea, tremors, anorexia, increase of the liver function parameters and fever. The most frequent adverse events unrelated to treatment were headache, alopecia, seizures and radiodermitis. Overall, serious adverse events included headache, vomiting, seizures, brain edema, pneumonia, hemiparesis, motor defects, disorientation, respiratory depression and intracranial hypertension. All serious adverse events were attributed to the natural course of the disease and the neurological worsening associated with HGG. None of the serious adverse events were attributed to nimotuzumab. Table 2 summarizes the most frequent unrelated and related toxicities for both treatment groups.

**Table 2:** Most frequent unrelated and related adverse events after treatment with irradiation plus nimotuzumab or placebo

**Adverse event**	**Placebo arm**	**Nimotuzumab arm**
	**Number of events**	**Number of events**
**Unrelated (Not related and unlikely)**		
Headache	42	17
Seizures	16	6
Dry radiodermitis	6	5
Fever	6	
Asthenia	5	4
Liver function tests alterations	7	5
Alopecia	4	7
**Possibly, probably or definitively related with nimotuzumab**		
Liver function tests alterations		8
Nauseas		4
Tremors		3
Anorexia		3
Fever		2

None of these patients developed anti-idiotypic response against the murine residues of the humanized molecule.

Antitumor response was confirmed for 33 patients in the AA stratum (17 controls and 16 nimotuzumab treated subjects) and 20 patients in the GBM arm (9 controls and 11 antibody treated subjects). No significant differences were detected between the 2 groups in relation to overall response or disease control rate. Objective response was 59.25% for nimotuzumab and 53.84%, for the placebo arm. Disease control rate was 85.18% for the active drug group vs. 84.61%, in the placebo cohort.

The mean and median survival time for the intent to treat (ITT) population in the nimotuzumab cohort was 31.06 and 17.76 months, and 21.07 and 12.63 months for those patients treated with placebo and irradiation (HR=0,64). This difference was statistically significant according the Weibull parametric model (Weibull statistics, p = 0.032).

For AA patients, the mean and median survival time was 41.29 and 44.56 months, if they received nimotuzumab vs. 29.67 and 17.56 months for the control patients (Weibull statistics, p = 0.311). For the GBM patients, mean and median overall survival corresponded to 17.24 and 8.40 (nimotuzumab arm) vs. 9.84 and 8.36 months (placebo arm), respectively (Weibull statistics, p = 0.026).

**Figure 1:**
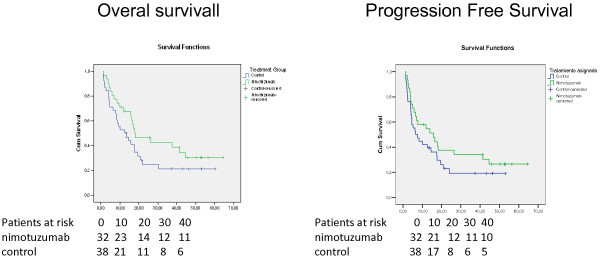
Overall survival and progression free survival of patients treated with radiotherapy and nimotuzumab or placebo.

**Figure 2:**
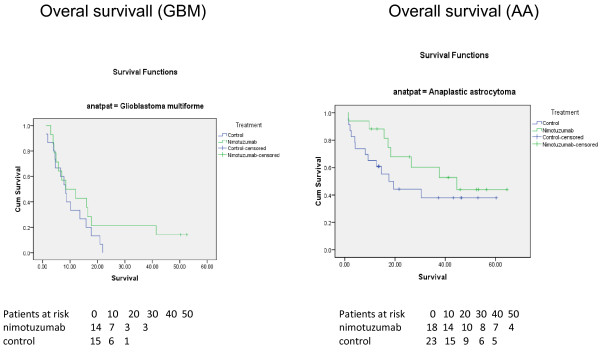
Overall survival of GBM and AA patients treated with radiotherapy and nimotuzumab or placebo.

PFS was evaluated as a secondary endpoint. In the ITT analysis, the mPFS was 15.73 months for nimotuzumab + RT and 6.5 months for the control arm. Overall survival and Progression free survival curves for the whole population are illustrated at Figure 1. Figure 2 presents the survival curves according histology.

## Discussion

Eventhough, chemo-radiotherapy is the standard of care for anaplastic astrocytomas and glioblastoma multiforme, patients’ prognosis remains very poor and the disease is still incurable. Thus, enrolling this patient population in clinical trials that evaluate new drug candidates is a very appealing strategy. Novel biologic therapies under clinical evaluation for patients with high grade glioma include dendritic cell vaccination, tyrosine kinase receptor inhibitors, farnesyl transferase inhibitors, viral-based gene therapy, oncolytic viruses, vascular endothelial growth factor inhibitors and Epidermal growth factor-receptor inhibitors [25,26]. This manuscript illustrates the results of combining irradiation and an anti-EGFR antibody in a double blind trial that complements the nimotuzumab add-on to temozolomide/irradiation study in GBM, which is underway under a German sponsorship in Europe [23].

Nimotuzumab was very well tolerated and the most frequent adverse reactions consisted of grade 1/2 infusion reactions. Remarkably, patients received a cumulative dose of 3300 mg, which is much higher than the dose administered in the previous Phase I trial (1200 mg) and is probably the highest cumulative antibody dose ever administered to glioma patients. After 16 doses of nimotuzumab, there was no increasing toxicity with repeated treatment. Radiation associated toxicity was not exacerbated by the antibody. As reported in all previous studies, nimotuzumab did not induce skin rash [11-13,20,27] and [28]. No anti-idiotypic response was detected, confirming nimotuzumab low immunogenicity.

Several EGFR inhibitors have been used to treat HGG patients [29-42]. Cetuximab was administered as monotherapy to recurrent glioma patients with an acceptable safety profile [30]. The most frequent adverse events have been acne-like rash (folliculitis/dermatitis) together with xerodermia, paronichia, fissures at the hands and/or feet, dermatitis of the eyelids, and increased facial hair growth [30]. Alternatively, small tyrosine kinase inhibitors have been used to treat recurrent or newly diagnosed glioma patients. More than 10 clinical trials using erlotinib or gefitinib, have been reported [31-42]. Globally, both drugs were well tolerated, being diarrhea and rash the most common toxicity. The majority of these events were mild or moderate, while grade 3 or higher events were reported in one-third of the patients receiving erlotinib in the recurrent scenario [31-36]. Strikingly, one trial evaluating the combination of temozolomide, radiotherapy and erlotinib was discontinued due to unacceptable toxicity [39].

Regarding clinical outcome, patients achieved a significant improvement in overall survival, if they received nimotuzumab and irradiation. However, this result should be interpreted with caution since even though no significant differences were detected between the 2 groups regarding the most important prognostic factors, more patients with poorer KPS and no debulking surgery were included to the control group.

Nimotuzumab has been evaluated before in combination with irradiation and temozolimide for the treatment of newly diagnosed GBM patients. In a trial conducted at 11 hospitals in Germany, patients were randomized to arm A (nimotuzumab/RT/TMZ) versus arm B (RT/TMZ). Results demonstrated a median overall survival (MOS) of 22.3 and 19.6 months for groups A and B that were comparable with the results of Hegi of 21.7 (RT/TMZ) vs. 15.3 months (RT) for patients with methylated MGMT [24], and better than Stupp’s of 14.6 (RT/TMZ) and 12.1 months (RT) [3] or Hegi, for patients with unmethylated MGMT of 12.7 (RT/TMZ) vs. 11.8 months (RT) [43]. Patients with non-methylated MGMT derived the greatest benefit after treatment with nimotuzumab: MOS, 19.6 months (arm A) vs 15.0 months (Arm B) [23]. The authors concluded that nimotuzumab shows a clear trend towards efficacy in MGMT non-methylated glioblastoma patients together with an excellent safety profile [23]. Our finding is comparable to the results of the German Phase III study, where the combination of nimotuzumab/RT/TMZ showed the greatest advantage over RT/TMZ in the subset of patients with non-methylated MGMT, who are resistant to the alkylating agent, via direct DNA repair [43]. Nimotuzumab didn’t significantly improve the rates of objective response or disease control. However, it increases PFS and overall survival, demonstrating its predominant cytostatic effect and its role in controlling the tumor progression rate.

Overall, patients achieved a lower survival than reported for AA and GBM, particularly if treated with placebo. This poor outcome can be explained by the baseline characteristics of the patient population: 29 patients (41.4%) were older than 50, 20 patients (28.6%) had a KPS of 60 or 70, 18 patients (25.7%) had just a biopsy, while only 7 patients (10%) got a gross tumor resection. Lower KPS, lack of debulking surgery and older ages are strong predictors of poor outcome, according the recursive portioning analysis proposed by RTOG and validated by EORTC [44,45].

So far, other EGFR antagonists have resulted in limited clinical activity in glioma patients [29-42]. Cetuximab has a low single-agent activity in patients with recurrent HGG [30]. Furthermore, erlotinib, when used in the recurrent setting has shown to be marginally beneficial [31-37]. For the newly diagnosed patients, erlotinib co-administered with radiotherapy and temozolomide was not efficacious [39]. We speculate that the lack of efficacy of other EGFR antagonists might be associated with reduced drug exposure. Treatment with cetuximab, erlotinib and gefitinib were maintained as long as there were no unacceptable safety concerns or until disease progression. Since these EGFR antagonists can induce severe acne-like rash toxicity, hypomagnesia and diarrhea, toxicity might have prevented protracted therapy.

## Conclusions

In this randomized, double blind, placebo-controlled trial, nimotuzumab continues to show an excellent safety profile (consistent with an international pharmacovigilance data base of 17,451 patients), and survival benefit in patients with high grade glioma in combination with irradiation.

## Abbreviations

AA: Anaplastic Astrocytoma; CT scan: Computer Tomography; CTC: Common Toxicity Criteria; EGFR: Epidermal Growth Factor Receptor; EORTC: European Organization for Research and Treatment of Cancer; GBM: Glioblastoma Multiforme; HGG: High Grade Glioma; KPS: Karnofsky Performance Status; MOS: Median overall survival; MRI: Magnetic Resonance Image; OS: Overall survival; PFS: Progression Free Survival; RT: Radiotherapy; RTOG: Radiation Therapy Oncology Group; TMZ: Temozolomide.

## Competing interests

All authors declare that they have no competing interests.

## Author’s contributions

MTS was the principal investigator of the clinical trial; JCS, JF, JV, NQ, SS, RD and CT were the principal investigators of the 7 other hospitals that took part in the multicentric study, JA and JJM and MC were the radiotherapists that irradiated the patients, MGG and JAM were the clinical research assistants that conducted the study, PLL and JB made the statistical analysis, NC evaluated the HAMA the response, FB made significant contributions to the elaboration and revision of the manuscript and TC was the project leader. All authors reviewed and approved the final version of the manuscript prior to its submission for publication

## Author’s information

Patricia Lorenzo Luaces and Tania Crombet are employees of the Center of Molecular Immunology, the research institution that patented and manufactures nimotuzumab. Ferdinand Bach works for Oncoscience AG, Wedel, a company that owns nimotuzumab marketing rights in Europe. The present study was sponsored by the Center of Molecular Immunology, Havana, Cuba and The Cuban Ministry of Health.

## Pre-publication history

The pre-publication history for this paper can be accessed here:

http://www.biomedcentral.com/1471-2407/13/299/prepub
